# First report of *Cryptosporidium canis* in farmed Arctic foxes (*Vulpes lagopus*) in China

**DOI:** 10.1186/s13071-016-1396-6

**Published:** 2016-03-03

**Authors:** Xiao-Xuan Zhang, Wei Cong, Jian-Gang Ma, Zhi-Long Lou, Wen-Bin Zheng, Quan Zhao, Xing-Quan Zhu

**Affiliations:** State Key Laboratory of Veterinary Etiological Biology, Key Laboratory of Veterinary Parasitology of Gansu Province, Lanzhou Veterinary Research Institute, Chinese Academy of Agricultural Sciences, Lanzhou, Gansu Province 730046 PR China; College of Animal Science and Technology, Jilin Agricultural University, Changchun, Jilin Province 130118 PR China

**Keywords:** *Cryptosporidium canis*, Prevalence, Arctic fox, China

## Abstract

**Background:**

*Cryptosporidium* is an important genus of enteric zoonotic parasites, which can infect a wide range of animals including foxes. Little information is available concerning the prevalence and molecular characterisation of *Cryptosporidium* spp. in farmed Arctic foxes (*Vulpes lagopus*) in China. Thus, the objective of the present study was to investigate the prevalence of *Cryptosporidium* spp. in Arctic foxes in China using nested PCR amplification of the small subunit ribosomal RNA (SSU rRNA) gene.

**Findings:**

The overall prevalence of *Cryptosporidium* spp. in Arctic foxes was 15.9 % (48/302), with 12.9 % in male (18/139) and 18.4 % in female (30/163) foxes, respectively. The prevalence in different farms varied from 0 to 31.43 %. The prevalence of infection in different age groups varied from 14.1 % to 19.0 %. Foxes from Hebei Province (7.8 %, 11/141) had a significantly lower *Cryptosporidium* spp. prevalence than those from Heilongjiang Province (22.9 %, 16/70) and Jilin Province (23.1 %, 21/91) (*P*= 0.0015). Sequence analysis of the SSU rRNA gene indicated that all the 48 isolates represented *C. canis*.

**Conclusions:**

This is the first report of *C. canis* infection in farmed Arctic foxes in China, which also provides foundation data for preventing and controlling *Cryptosporidium* infection in foxes, other animals and humans.

## Findings

### Background

Cryptosporidiosis is caused by species of *Cryptosporidium*, important zoonotic protozoan parasites [1–3]. *Cryptosporidium* spp. not only have a cosmopolitan distribution but can also infect a wide range of animals including foxes [1, 4]. Humans and animals are often infected through faecal-oral route and infection can result in acute or chronic diarrhea and even death [3, 5]. So far, more than 17 *Cryptosporidium* species/genotypes, such as *C. andersoni*, *C. parvum*, *C. hominis*, *C. meleagridis*, *C. felis*, *C. canis*, *C. muris*, *C. suis* and *Cryptosporidium* sp. deer genotype, have been identified in humans [6–10], but only *C. parvum*, *Cryptosporidium* sp. muskrat genotype II and *C. canis* have been found in foxes [6, 11, 12].

The Arctic fox (*Vulpes lagopus*) is common in the Arctic regions [4], and has been imported to China from the former Soviet Union in the 1950s [13]. In China, with the improvement of living standards, Arctic foxes were commonly raised by farmers to provide furs for humans. More importantly, because of the close  relationship between farmed foxes and humans, foxes can transfer indirectly or directly many pathogens to humans, such as *T. gondii* [14]. Some studies concerning *Cryptosporidium* spp. infections in foxes have been reported [4, 6, 12, 15], but no such information about *Cryptosporidium* spp. prevalence in foxes is available in China. The objective of the present study was to estimate the prevalence of *Cryptosporidium* infection in farmed foxes in China, for the first time.

## Methods

### Ethics statement

This study was approved by the Animal Ethics Committee of Lanzhou Veterinary Research Institute, Chinese Academy of Agricultural Sciences (Approval No. LVRIAEC2013010). The Arctic foxes from which the faeces were collected, were handled in accordance with good animal practices required by the Animal Ethics Procedures and Guidelines of the People’s Republic of China.

### Specimen collection

A total of 302 faecal samples from 91 foxes in Jilin Province, from 70 foxes in Heilongjiang Province, and from 141 foxes from Hebei Province, were collected in 2014. All foxes were in good health during the sampling time. Fresh faecal samples were collected from each animal using sterile gloves immediately after the defecation onto the ground and transported to the laboratory. Information regarding geographical origin, gender and age of the foxes were acquired by a questionnaire.

### DNA extraction and PCR amplification

Genomic DNA was extracted from faeces using an EZNAR Stool DNA kit (OMEGA, USA) following the manufacturer’s instructions and stored at -20 °C until PCR analysis. *Cryptosporidium* species/genotypes were identified by nested PCR amplification of the small subunit ribosomal RNA (SSU rRNA) gene [3]. Every amplification included positive and negative controls. Amplification products were visualised on  1.5 % agarose gels containing GoldView™ (Solarbio, China).

### Sequencing and phylogenetic analyses

Positive secondary PCR products from foxes were sequenced by the Genscript Company (Nanjing, China). *Cryptosporidium* species/genotypes were identified by comparison with reference sequences using BLAST (http://www.ncbi.nlm.nih.gov/BLAST/) and computer program Clustal X 1.83. Phylogenetic relationships of *Cryptosporidium* spp. were reconstructed using Neighbour-Joining (NJ) method implemented in  Mega 5.0 (Kimura 2-parameter model, 1,000 replicates). All representative nucleotide sequences obtained were deposited in the GenBank under accession numbers KU215430-KU215436.

### Statistical analysis

The variation in *Cryptosporidium* spp. prevalence (*у*) in foxes in relation to geographical location (*x*1), gender (*x*2) and age (*x*3) were analysed by *χ*2 test using SAS version 9.1 (SAS Institute Inc., USA). Using multivariable regression analysis each of these variables was included in the binary Logit model as an independent variable. The best model was identified  by Fisher’s scoring algorithm. All tests were two-sided. Results were considered statistically significant at *P *< 0.05. Odds ratios (ORs) with 95 % confidence intervals (95 % CI) were also calculated.

## Results and discussion

**Fig. 1:**
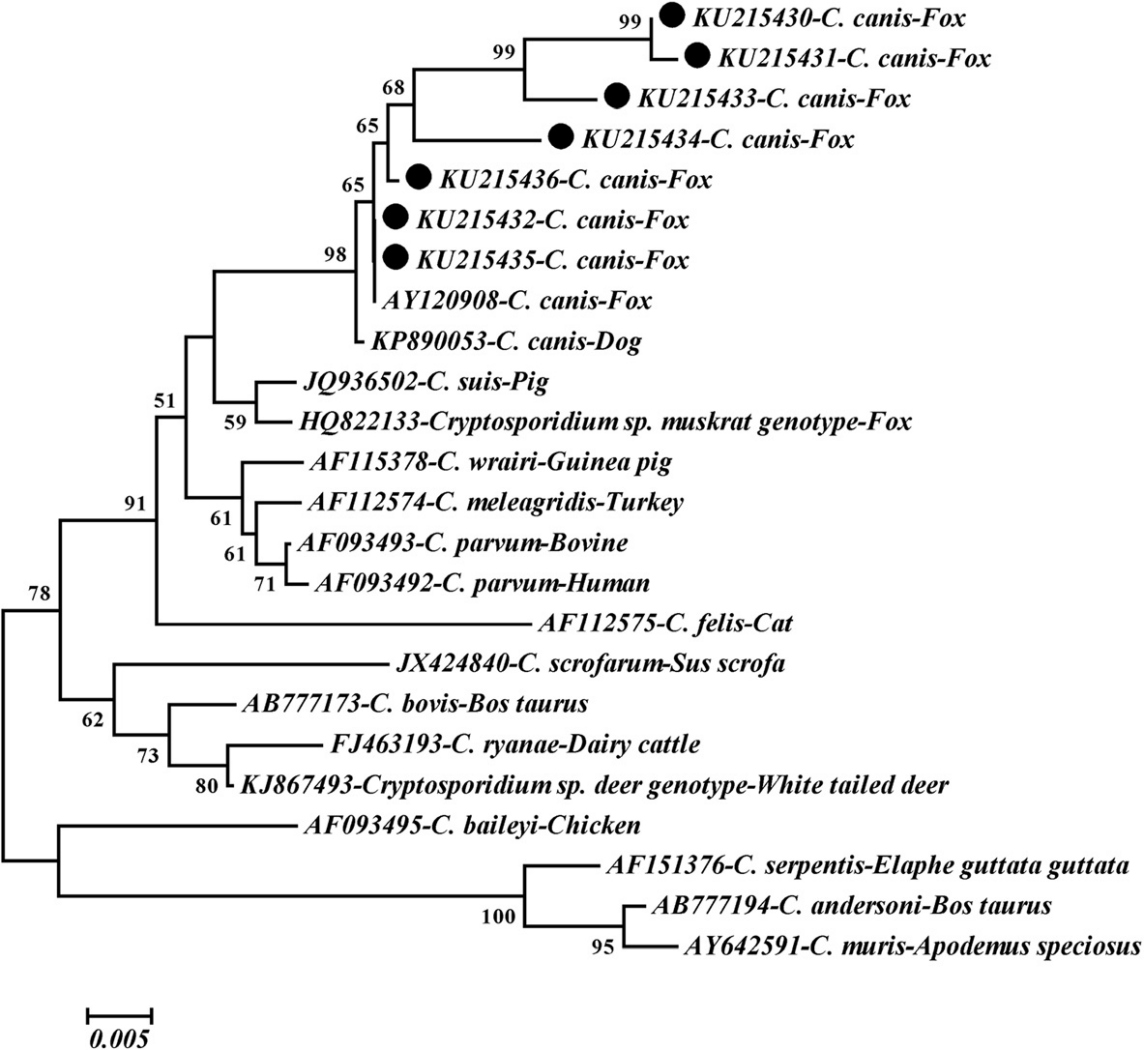
Phylogenetic analysis of *Cryptosporidium canis* using Neighbour-Joining (NJ) method based on sequences of the small subunit ribosomal RNA (SSU rRNA) gene. Bootstrap values >50 % are shown. Isolates of *C. canis* identified in the present study are indicated by a solid circle

A total of 48 out of 302 Arctic foxes (15.9 %) were tested *Cryptosporidium*-positive by nested PCR amplification of the SSU rRNA gene (Table 1). The prevalence in different farms varied from 0 to 31.4 % (data not shown). *Cryptosporidium* spp. prevalence was 14.1 % (9/64) in pre-weaned foxes, 15.6 % (28/180) in young foxes, and 19.0 % (11/58) in adult foxes (Table 1). The prevalence in different regions varied between 7.8–23.1 % (Table 1). Moreover, female foxes (18.4 %, 30/163) had a higher *Cryptosporidium* prevalence than males (12.9 %, 18/139), although the differences were not significant (Table 1). Sequence analysis of the SSU rRNA gene indicated that all of the 48 isolates represented *C. canis* (Fig. 1).

**Table 1:** Prevalence of *Cryptosporidium* *canis* in farmed foxes in Jilin, Heilongjiang and Hebei Provinces, northern China

Factor	Category	No. of tested	No. of positive	Prevalence (%) (95 % CI)	Odds Ratios (OR) (95 % CI)	*P*-value
Region	Hebei Province	141	11	7.8 (3.4–12.2)	Reference	0.0015
	Heilongjiang Province	70	16	22.9 (13.0–32.7)	3.5 (1.5–8.0)	
	Jilin Province	91	21	23.1 (14.4–31.7)	3.6 (1.6–7.8)	
Gender	Male	139	18	12.9 (7.4–18.5)	Reference	0.1962
	Female	163	30	18.4 (12.5–24.4)	1.5 (0.8–2.9)	
Age	Pre-weaned	64	9	14.1 (5.6–22.6)	Reference	0.7463
	Young	180	28	15.6 (10.3–20.9)	1.1 (0.5–2.5)	
	Adult	58	11	19.0 (8.9–29.1)	1.4 (0.6–3.8)	
Total		302	48	15.9 (11.8–20.0)

In the present study, the overall *Cryptosporidium* spp. prevalence was 15.9 % (95 % CI 11.8-20.0) (Table 1), which was higher than that in wild Arctic foxes in the central Canadian Arctic (9 %) [4], wild foxes in wetlands adjacent to the Chesapeake Bay, USA (8 %) [6], wild red foxes in Ireland (1.6 %) and Warwickshire, UK  (8.7 %) [12],  and Norway (2.2 %) [16], but lower than that in red foxes in the Slovak Republic (38.7 %) [11]. These differences might be related to the detection methods, age distribution of the samples, the timing of sample collection, sample sizes and geo-ecological conditions in the investigation regions.

The effects of geographical location, gender and age were analysed using univariate analysis. The impacts of multiple variables on the prevalence of *C. canis* were evaluated by forward stepwise logistic regression analysis using Fisher’s scoring technique. In the final model, only one variable had a significant effect, described by the equation *y* = 0.5964*x*1 + 0.4646. Region of origin has a positive effect on the risk of *C. canis* ( OR =1.8, 95 % CI 1.3–2.6). Foxes collected from the Jilin Province (23.1 %. OR = 3.6, 95 % CI 1.6–7.8) and Heilongjiang Province (22.9 %, OR = 3.5, 95 % CI 1.5–8.0) were found to be more susceptible than those collected from the Hebei Province (7.8 %, 95 % CI 3.4-12.2, *P* = 0.0015) (Table 1).

Three *Cryptosporidium* species/genotypes (*C. parvum*, *C. canis* and *Cryptosporidium* sp. muskrat genotype II) have been found in foxes [4, 6, 12, 15]. Of these, *C. parvum* and *C. canis* have also been reported in humans [17, 18] suggesting that foxes could be a potential resource for humans acquiring cryptosporidiosis. In the present study, all of the 48 *Cryptosporidium*-positive samples represented *C. canis* (Fig. 1), which was similar to previous studies showing that *C. canis* is more prevalent in foxes [4, 6, 11, 16]. However, probably due to the smaller sample sizes, *C. parvum* and *Cryptosporidium* sp. muskrat genotype II were not found in the present study.

## Conclusions

The results of the present study indicated the existence (15.9 %, 48/302) of *C. canis* infections in farmed Arctic foxes in northern China. Logistic regression analysis indicated that region was the significant risk factor shown by this study for *Cryptosporidium* spp. infection in the foxes examined. The data could provide a foundation for the prevention and control of *Cryptosporidium* spp. infections in foxes, other animals and humans.
